# Imbalance of Th17/Treg cells in pathogenesis of patients with human leukocyte antigen B27 associated acute anterior uveitis

**DOI:** 10.1038/srep40414

**Published:** 2017-01-16

**Authors:** Zhenchao Zhuang, Yuqin Wang, Gejing Zhu, Yunfeng Gu, Liping Mao, Meng Hong, Yali Li, Meiqin Zheng

**Affiliations:** 1 Division of Clinical Lab, Eye Hospital of Wenzhou Medical University, Wenzhou 325035, Zhejiang, China; 2 School of Laboratory Medicine and Life Science, Wenzhou Medical University, Wenzhou 325025, Zhejiang, China; 3 Department of Ophthalmology, Eye Hospital of Wenzhou Medical University, Wenzhou 325035, Zhejiang, China

## Abstract

Th17 and regulatory T cells, involved in the pathogenesis of several autoimmune diseases, are new lineages of CD4+ T helper cells. However, the role of their imbalance in human leukocyte antigen B27-associated acute anterior uveitis has not been elucidated. In our study, the percentages of Th17 and Treg cells, their molecular markers and related factors in peripheral blood of patients and healthy controls were measured by flow cytometry, real-time RT-PCR and ELISA. We observed a remarkable increase of CD4+ and CD4+IL-17+ T cells in peripheral blood of patients compared to controls. The molecular markers and related factors of Th17 cell were also showed a distinct elevation. Interestingly, we observed an obvious decrease of CD4+CD25+Foxp3+ T cells and Foxp3 mRNA level in patients. The ratio of Th17/Treg in patients was dramatically higher than controls. Moreover, the ratio of Th17/Treg cells had a more significantly positive correlation with the disease activity score than Th17 cells whereas Treg cells had a negative correlation. Our findings demonstrated a distinct increase of Th17 cells and a significant decrease of Treg cells in patients compared to controls. The imbalance of Th17 and Treg cells may play a vital role in the pathogenesis of the disease.

Human leukocyte antigen B27-associated acute anterior uveitis (HLA-B27-associated AAU, here after referred to as AAU) is defined as anterior segment intraocular inflammation with the positive of HLA-B27 antigen, which is a human class I major histocompatibility complex (MHC). It is the most common form of anterior uveitis, accounting for 18–32% of all cases of anterior uveitis^1^. The typical phenotypes of HLA-B27-associated AAU are acute onset of unilateral alternating, non-granulomatous acute anterior uveitis, being characterized by significant cellular and protein extravasation into the aqueous humor including fibrin and hypopyon in the anterior chamber, high tendency for recurrences, and significant association with other HLA-B27-related diseases such as seronegative spondyloarthropathies (SpA)^2^. Considering the high frequency of recurrent attacks and sever ocular complication, AAU is regarded as a potentially sever threat to version.

It is clear from the evidence of epidemic logical and experimental studies that both genetic and environmental factors are critically important in the pathogenesis of AAU. It is well known that the immune factors play a primary role in the development and progression of the disease, but their precise mechanisms have not yet been elucidated. Recently, lymphocytes have received considerable attention for the roles in autoimmune diseases, and there is more and more evidence indicate that T lymphocytes play pivotal roles in the pathogenesis of autoimmune uveitis^3^.

Th17 cells are a distinct lineage of CD4^+^ T helper cells that are new described recent years, which excited us for bring new realization of immunoloregulation and host defense. Th17 cells are characterized by secreting multiple cytokines such as IL-17A, IL-17F, IL-6 and IL-22, which have been involved in the pathogenesis of several T cell mediated autoimmune diseases, such as rheumatoid arthritis and multiple sclerosis^4^,^5^,^6^,^7^. Several reports pointed out that Th17 cells were presented in human peripheral blood mononuclear cells (PBMC) of uveitis. Their amount increased during active uveitis and decreased following treatment, suggesting a mechanism by which Th17 may contribute to ocular pathology. However, the results were extremely rare when they were accurate to AAU.

Regulatory T cells (Treg cells) are another cluster of CD4^+^ T helper cells, which are characterized by production of TGF-β and IL-10 and play an important role in regulating of self-tolerance and maintenance of T-cell homeostasis^8^,^9^. It was reported that the dysfunction of Treg cells are involved in certain human autoimmune diseases, such as experimental autoimmune encephalomyelitis (EAE) and rheumatoidarthritis^10^,^11^.

Furthermore, it is reported that ablating Treg in mice could lead to sever autoimmune disease^12^. More importantly, some reports indicated that therapy of autoimmune uveitis results in the increase of T-regulatory lymphocyte population and restoration of their functional state^13^. According to these proves, it is reasonable to hypothesize that the balance of Th17/Treg is important for immunological tolerance of the hosts and autoimmune disease. Therefore, we conducted to explore the role of imbalance of Th17/Treg in the pathogenesis of AAU.

## Results

### Participants

Patients with AAU were recruited between January 2012 and June 2013 from the Affiliated Eye Hospital of Wenzhou Medical University. Twenty AAU patients were enrolled in this study. Twenty age- and sex-matched healthy individuals were set as control (Table 1).

### The proportion of CD4^+^ and CD4^+^IL-17^+^ T cells increased in PBMC of HLA-B27 -associated AAU patients

**Figure 1:**
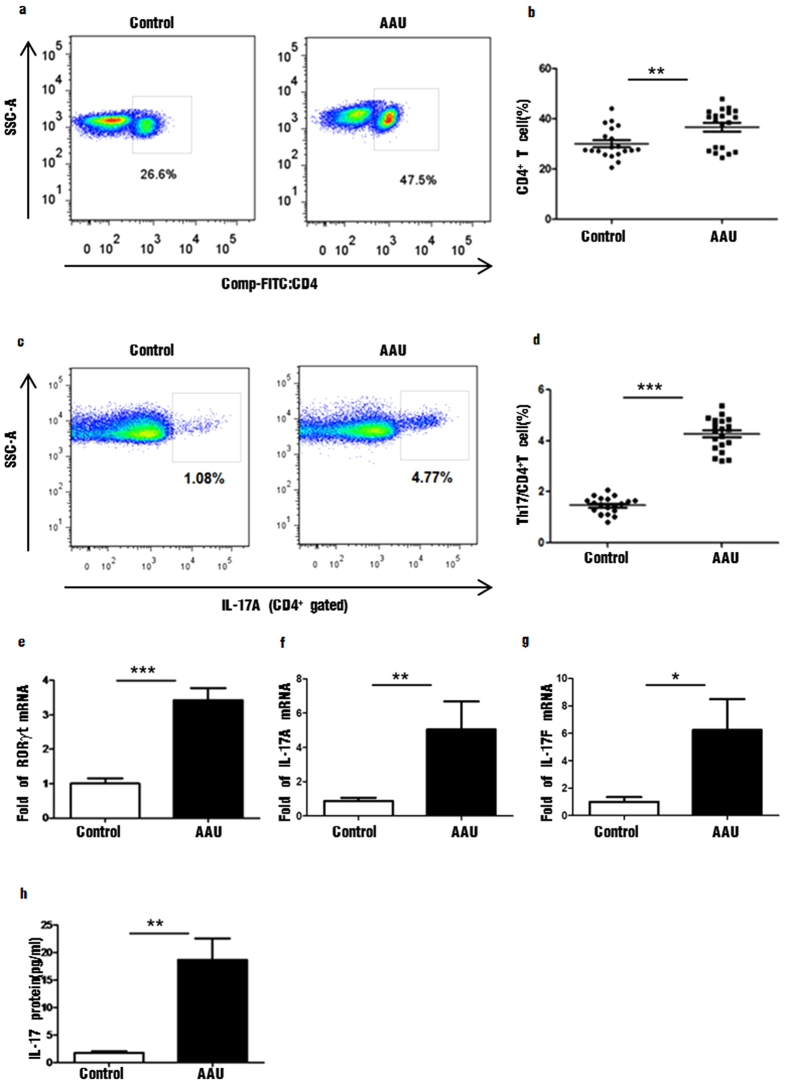
The distribution of CD4^+^, CD4^+^ IL-17^+^ cells and Th17 specific transcription factors and cytokines in peripheral blood of patients with HLA-B27-associated AAU and controls. (**a**,**b**) CD4^+^ cells in control group and patients with HLA-B27-associated AAU; (**c**,**d**) CD4^+^IL-17A^+^ cells in control group and patients with HLA-B27-associated AAU (**e–g**). The mRNA levels of RORγt, IL-17A, IL-17F expressed by PBMC in control group and patients with HLA-B27-associated AAU (**h**). The protein concentration of IL-17 in the serum of control group and patients with HLA-B27-associated AAU. Data represent means ± SDs. Data were analyzed using Student’s t-test. Error bars represent s.e.m. *P < 0.05; **P < 0.01; ***P < 0.001 each control group vs HLA-B27-associated AAU patients. (AAU = acute anterior uveitis).

According to flow cytometric analysis, the percentage of CD4^+^ T cells in PBMC was dramatically increased in AAU patients (36.60% ± 1.72, ranged 24.50–47.90%), compared to healthy controls (30.05% ± 1.36, ranged 20.50–39.10%) (*P* < 0.01) (Fig. 1. Top left and right). The percentage of CD4^+^IL-17^+^ T cells was substantially elevated in patients (4.27% ± 0.15, ranged 3.2–5.38%), compared to healthy individuals (1.47% ± 0.07, ranged 0.82–2.07%) (*P* < 0.001) (Fig. 1. Bottom left and right).

### The level of Th17 specific transcription factors and cytokines were increased in AAU patients

**Figure 2:**
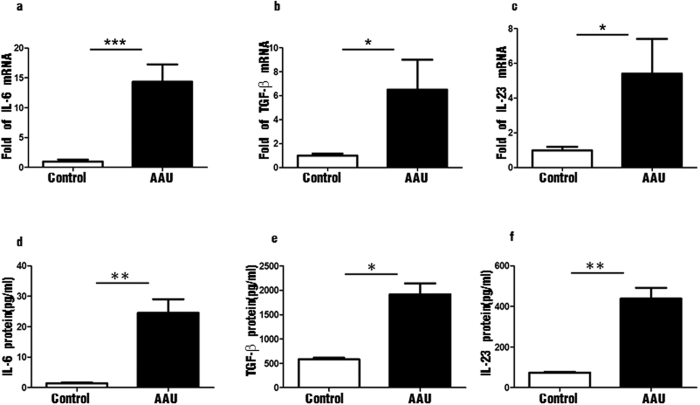
The distribution of Th17 associated transcription factors and cytokines in peripheral blood of patients with HLA-B27-associated AAU and controls. (**a–c**) The mRNA levels of IL-6, TGF-β and IL-23 expressed by PBMC in control group and patients with HLA-B27-associated AAU; (**d–f**). The protein concentrations of IL-6, TGF-β and IL-23 in the serum of control group and patients with HLA-B27-associated AAU. Data represent means ± SDs. Data were analyzed using Student’s t-test. Error bars represent s.e.m. *P < 0.05; **P < 0.01; ***P < 0.001 each control group vs HLA-B27 AAU patients. (AAU = acute anterior uveitis).

We examined the specific transcription factors of Th17 cells by real-time PCR. Retinoic acid-related orphan receptor γ thymus (ROR-γt) encoded by gene Rorc was identified as the master transcription factor of Th17 cells. The mRNA level of ROR-γt was dramatically increased in peripheral blood of AAU patients, compared with healthy controls (*P* < 0.001) (Fig. 2. Top left). IL-17A (also known as IL-17) and IL-17F are the founding members of the IL-17 cytokine family. The genes encoding IL-17A and IL-17F are localized in the same chromosomal region in mice and in humans. The mRNA level of IL-17A and IL-17F in peripheral blood of patients with AAU was significant increased, compared to the control group (*P* < 0.001, *P* < 0.05) (Fig. 2 Top right and Bottom left). We also determined the protein concentration of IL-17 in AAU patients and healthy controls by ELISA according to the manufacturer’s protocol. The protein concentrations of IL-17A (18.70 ± 3.90 pg/ml) in serum of AAU patients were significantly higher than controls (1.77 ± 0.30 pg/ml) (*P* < 0.01) (Fig. 2. Bottom right).

### The inducing factors of Th17 cells were elevated in patients with HLA-B27AAU

**Figure 3:**
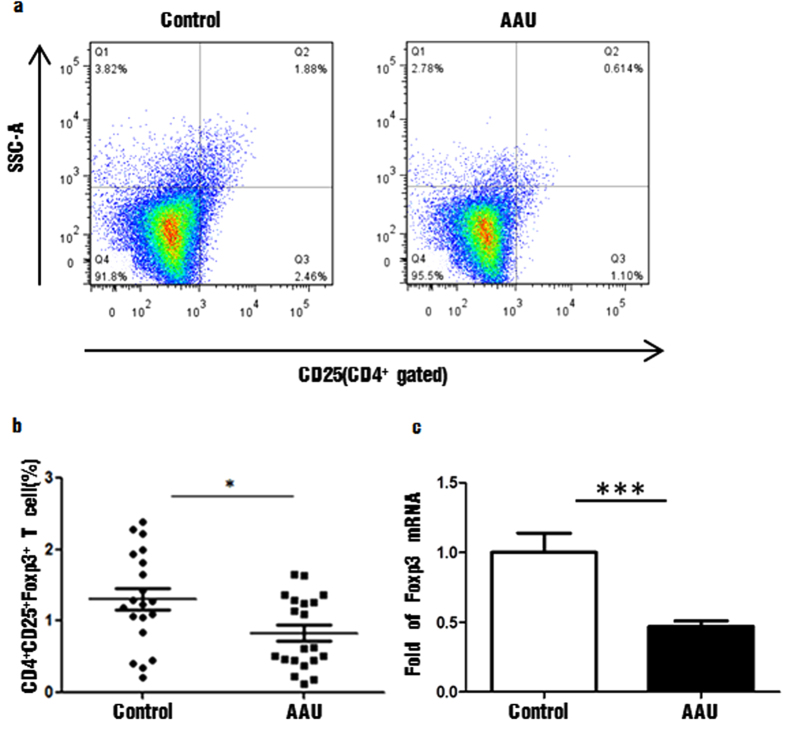
The distribution of CD4^+^CD25^+^Foxp3^+^ cells and its specific transcription factor in peripheral blood of patients with HLA-B27-associated AAU and controls. (**a**,**b**) CD4^+^CD25^+^Foxp3^+^ cells in control group and patients with HLA-B27-associated AAU (**c**). The mRNA level of Foxp3 expressed by PBMC in control group and HLA-B27-associated AAU patients. Data represent means ± SDs. Data were analyzed using Student’s t-test. Error bars represent s.e.m. *P < 0.05; **P < 0.01; ***P < 0.001 each control group vs HLA-B27-associated AAU patients. (AAU = acute anterior uveitis).

The differentiation of Th17 cells from naive CD4^+^ T cells is regulated by cytokines. Transforming growth factor-β (TGF-β) plus IL-6 initiate Th17 differentiation both *in vitro* and *in vivo*^14^,^15^, and IL-23 expands and stabilizes previously differentiated Th17 cells. The levels of IL-6, TGF-β and IL-23 mRNA were all increased in peripheral blood of AAU patients compared with healthy controls. (*P* < 0.001, *P* < 0.05, *P* < 0.05) (Fig. 3. Top left, center and right). The protein concentrations of IL-6 (24.55 ± 4.50 pg/ml), TGF-β (2053.00 ± 176.00 pg/ml), IL-23 (438.22 ± 53.28 pg/ml) in serum of AAU patients were significantly higher than controls as well (1.40 ± 0.29 pg/ml, 1366.10 ± 192.00 pg/ml, 78.12 ± 3.54 pg/ml, *P* < 0.001, *P* < 0.05, *P* < 0.001) (Fig. 3. Bottom left, center and right).

### The percentage of CD4^+^CD25^+^Foxp3^+^ Treg cells and their specific transcription factor were decreased in HLA-B27 AAU patients

**Figure 4:**
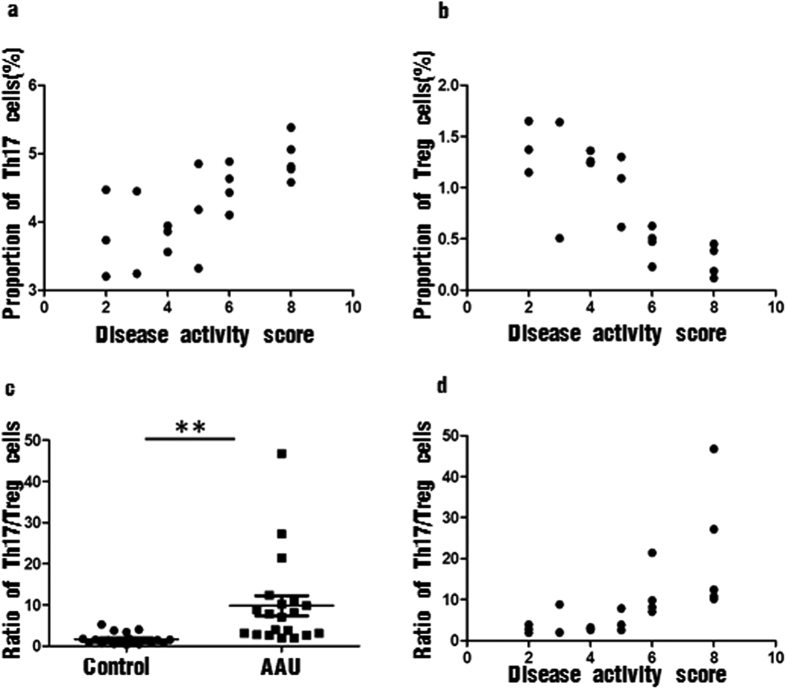
Correlation analysis between the proportion of the two CD4^+^ T cell subsets and their ratio in peripheral blood of patients with HLA-B27-associated AAU and disease activity score. (**a**) Correlation analysis between the proportion of Th17 cells and disease activity score (R = 0.715, *P* < 0.001); (**b**) Correlation analysis between the proportion of Treg cells and disease activity score (R = −0.842, P < 0.001). (**c**) The scatter graphs to show the ratio of Th17/Treg cells in control group and patients with HLA-B27-associated AAU; (**d**) Correlation analysis between the ratio of Th17/Treg cells in peripheral blood of patients with HLA-B27-associated AAU and disease activity score. (R = 0.805, P < 0.001). (Spearman’s rank correlation, n = 20) (AAU = acute anterior uveitis).

Percentage of Treg cells refers to the ratio of CD4^+^CD25^+^Foxp3^+^ cells to the total amount of CD4^+^ T lymphocytes in peripheral blood mononuclear cells. The percentage of CD4^+^CD25^+^Foxp3^+^ cells was significantly decreased in PBMC of AAU patients (0.83% ± 0.11, ranged 0.11–1.65%) than healthy controls (1.31% ± 0.35, ranged 0.21–2.39%) (*P* < 0.01) (Fig. 4. Top and Bottom left). We also examined the specific transcription factors of Treg cells by real-time PCR. Forkhead box protein P3 (Foxp3) was the specific transcription factor of Treg cell. The level of Foxp3 mRNA was markedly decreased in AAU patients compared with healthy controls (P < 0.01) (Fig. 4. Bottom right). The results of real-time PCR were consistent with flow cytometry analysis above.

### Correlation analysis between the proportion of Th17 and Treg cells in peripheral blood of patients with AAU and disease activity score

As Fig. 4. Top left shows (Fig. 4. Top left), the proportion of Th17 cells in peripheral blood of patients with AAU had a positive correlation with the disease activity score (R = 0.715, P < 0.001). Meanwhile, a negative correlation existed between the expression of Treg cells and the disease activity score (R = −0.842, *P* < 0.001) (Fig. 4. Top right).

### Th17/Treg imbalance was observed in HLA-B27 AAU patients

We observed an obvious imbalance of the mean ratio of Th17 to Treg in PBMC of AAU patients. The ratio of Th17 to Treg was markedly increased in patients with AAU (9.80 ± 2.44, ranged 1.93–46.78), compared with healthy controls (1.69 ± 0.30, ranged 0.34–5.24) (*P* < 0.01) (Fig. 4. Bottom left). More importantly, the ratio of Th17/Treg cells in peripheral blood of patients with AAU had a more significantly positive correlation with the disease activity score (R = 0.805, *P* < 0.001) than Th17 cells (R = 0.715, P < 0.001) (Fig. 4. Bottom right).

## Discussion

Since the discovery of Th17 and Treg cells, more and more efforts have been devoted to determining their biologic functions in various diseases of humans. Th17 is a pro-inflammatory T helper cell subset discovered in recent years. It is believed to be involved in the physiological or pathological processes in various autoimmune and inflammatory diseases mainly by secreting IL-17A and other cytokines, such as IL-6, IL-21 and IL-22^16^. As for uveitis, there were few reports about the role of Th17 cells in HLA-B27-associated AAU. However, in other subtypes of uveitis such as Vogt-Koyanagi-Harada disease and Behcet disease, the frequencies of IL-17-producing T cells from PBMCs were significantly upregulated in the active period of uveitis^17^,^18^. In our study, the expression of Th17 cells, Th17-specific transcription factor (ROR-γt, IL-17A and IL-17F mRNA level) and Th17-specific cytokines (IL-17A protein level) were all significantly increased in peripheral blood of AAU patients. More importantly, the proportion of Th17 cells in peripheral blood of patients had a positive correlation with the disease activity score. These results all suggest that Th17 cells may sustain inflammatory and Th17-mediate immune response may involve in the pathogenesis of AAU.

CD4^+^CD25^+^ Foxp3^+^ regulatory T (Treg) is another important T helper cell subset which can maintain self-tolerance and prevent the development of various inflammatory diseases. It functions by directly contacting effective immune cells and secreting anti-inflammatory cytokines, like interleukin (IL)-10 and transforming growth factor (TGF)-β. In human autoimmune uveitis, it was found a decrease in CD4^+^CD25^+^Foxp3^+^ T cells in active uveitis^19^. But in EAU, a useful tool for research human uveitis, the frequency of CD4^+^CD25^+^Foxp3^+^ T cells increased significantly^20^. In our study, the frequency of CD4^+^CD25^+^Foxp3^+^ Treg cells and the expression of Foxp3 mRNA were significantly decrease in AAU, which was consistent with previous studies in human uveitis. Results from correlation analysis showed that Treg cells and the disease activity score have the negative correlation, which suggested that Treg cells may contribute to the remission of the disease.

To further explore the mechanisms involved in the cytokines regulation of Th17 and Treg cells, real-time PCR and ELISA were performed to determine the levels of their relevant transcription factors and cytokines such as IL-6, TGF-β and IL-23. Upon antigen presentation by antigen-presenting cells (APCs), naive CD4^+^ T cells differentiate into any Th subset based on the cytokine milieu produced by the presenting APCs and surrounding mesenchymal cells. Initial studies suggested that IL-23 is the differentiation factor for Th17 cells, but IL-23 cannot act on naïve T cells. Later, TGF-β together with IL-6 was shown to initiate Th17 differentiation both *in vitro* and *in vivo*^14^,^15^, but its maintenance and phenotype depend on the IL – 23. It was surprised to see that TGF-β is required for the differentiation of Th17 cells in the presence of inflammatory cytokine IL-6 because TGF-β, an indispensable cytokine for the generation of Treg cells, had been recognized as an anti-inflammatory cytokine with a regulatory nature^21^. Although TGF-β (especially with addition of exogenous IL-2) upregulates Foxp3 and generates induced Treg cells, IL-6 inhibits the expression of Foxp3. This suggests that Treg and Th17 cells are developmentally related. In our study, we found that in the patients of AAU, the expression of TGF-β, IL-6 and IL-23 were significant increased in mRNA level. More importantly, the results of IL-6 showed a more significant difference than TGF-β and IL-6 in statistics. The changes of these cytokines were consistent with the change tendency of Th17 and Treg cells in peripheral blood. Taken together, the data suggested that although the expression of TGF-β was high in patients of HLA-B27 AAU, but the level of IL-6 was higher than TGF-β so that IL-6 inhibited the expression of Foxp3. Furthermore, the high expression of IL-23 further ensured the maintenance of the differentiation of Th17. In a word, the balance between the productions of pro- and anti-inflammatory cytokines is a key modulator of the development and balance of Th17 and Treg cells.

So far, limited information exists regarding the balance of Th17/Treg in patients with AAU. In the present study, we investigated a possible involvement of the imbalance of Th17/Treg cells in the pathogenesis of AAU in humans. The results showed a markedly higher of Th17 cells, Th17-related transcription factors and cytokines in the peripheral blood of AAU compared with healthy controls. Meanwhile, the number of CD4^+^CD25^+^Foxp3^+^ Treg cells and their specific transcription factor were significantly decreased. These results collectively suggested that an imbalance of Th17/Treg cells may be involved in the pathogenesis of AAU.

We also found that the percentage of CD4^+^IFN-γ^+^ and CD4^+^IL-4^+^ T cells in peripheral blood of HLA-B27-positive AAU patients was higher than that of the control group, but there was no statistically significant difference between the two groups (Supplementary Fig. S1. Top left and right, Supplementary Fig. S1. Centre left and right).

Interestingly, we found a group of IFN-γ and IL-17A double expressing Th17 cells (Supplementary Fig. S1. Bottom left and right). This is not the first time discovering IFN-γ and IL-17A double expressing Th17 cells in autoimmune diseases^22^,^23^, because many labs reported that they discovered those cells, no matter in human being’s blood or mice’ spleen. In our experiment, we did not test the pathogenic function of IFN-γ and IL-17A double expressing Th17 cells, but the proportion of those cells in the experimental group significantly increased compared with that of control group. Therefore, we believe that IFN-γ and IL-17A double expressing Th17 cells may be involved in the disease development, which would offer guiding significance by further understanding of the mechanism of Th cells in AAU.

As far as we know, there are no published studies on the imbalance of Th17 and Treg cells in patients of AAU. In our study, we calculated the ratio of Th17/Treg to observe the balance of Th17/Treg cells in patients of AAU and control group for the first time. We observed a dramatical increase of Th17/Treg ratio in the peripheral blood of AAU patients, and the ratio of Th17/Treg cells in patients had a more significantly positive correlation with the disease activity score than Th17 cells. These all indicated that the imbalance of Th17 and Treg cells may contribute to the pathogenesis of AAU and the ratio of Th17/Treg cells may be able to act as an indicator of disease activity.

## Conclusion

In sum, we observed a significant increase of Th17 cell percentage and its molecular markers in PBMC of patients compared with healthy controls whereas the proportion of Treg cell and its mark were markedly decreased. In addition, the inducing cytokines of Th17 cell were significantly increased in patients as well. More importantly, the ratio of Th17/Treg was dramatically increased compared to controls and correlation analysis showed a more significantly positive correlation with the disease activity score than Th17 cell. Our results suggest an important role of Th17/Treg imbalance in the development and progression of HLA-B27-assciated AAU and the ratio of Th17/Treg may be able to act as an indicator of disease activity. Further study on the underlying mechanism regulating the Th17/Treg balance may lead to a novel therapeutic strategy for HLA-B27-assciated AAU.

## Materials and Methods

All experiments were carried out in accordance with protocols approved by Ethics Committee in Eye Hospital of Wenzhou Medical University and Wenzhou Medical University.

### Study Population and Inclusion/Exclusion Criteria

This study was approved by the Ethics Committee of an institutional review board. Informed consent was obtained from all participants.

Patients with AAU were recruited between January 2013 and June 2014 from the Affiliated Eye Hospital of Wenzhou Medical University. The inclusion criteria: all carriers with an HLA-B27 antigen (Standard serologic methods were used for the detection of the HLA-B27 allele) and with an active intraocular inflammation (active anterior uveitis was defined by anterior chamber cells ≥1+); age from 20 to 50 years; not being treated naive for disease-modifying drugs, corticosteroids and biologics. Exclusion criteria comprised: all other types of uveitis; suspicion of any underlying ocular or systemic disease.

### Disease activity score

Following criteria was used to evaluate the disease activity of AAU patients^24^,^25^: keratic precipitates (one point), positive aqueous cells or Tyn (one point), conjunctival ciliary hyperemia (one point) and any synechia (one point). If the patient was accompanied by a complication such as cystoid macular edema, vitreous cells, secondary glaucoma or complicated cataract (one point).

### Detecting Th17 and Treg cells by flow cytometry

Ten milliliters of fasting blood were collected from the patients and the control group with Ethylene Diamine Tetraacetic Acid (EDTA) anticoagulant tubes. Mononuclear cells were isolated by density centrifugation over Ficoll-Paque Plus (GE, USA), and then the cell numbers were counted using the hemocytometer. According the results of cell counts, the concentration of cells were adjsuted to 2 × 10^6^ cells/ml. PBMC were cultured and stimulated in 24-well plates (2 × 10^6^ cells/well) containing complete RPMI 1640 medium (Gbico, USA) with 10% fetal calf serum (FCS, Biochrom AG, Berlin) immediately after isolation. The activation strategy was performed by follows: cells were stimulated for 4–6 h with phorbol 12-myristate 13-acetate (PMA) (50 ng/ml) and ionomycin (1 μg/ml) in the presence or absence of brefeldin A (BFA) (1 μg/ml) (all three from Sigma-Aldrich, USA). For analysis of Treg cells, PBMC (2 × 10^6^/ml) were aliquot into tubes without PMA and inomycin stimulation.

Fluorochrome-conjugated mAbs from eBioscience Pharmingen were used to detect the expression of CD4, CD25, IL-17A and Foxp3. FITC-labeled anti-human CD4 and PE-labeled anti-human IL-17A were used to examine Th17 cells. FITC-labeled anti-human CD4, PE-labeled anti-human CD25 and PE/Cy5.5-labeled anti-human Foxp3 were used to detect CD4^+^CD25^+^Foxp3^+^ Treg cells. After stimulated, PBMC (2 × 10^6^/mL) were collected into tubes, and surface stained for anti-CD4 at 4 °C for 30 min and treated with the Fix and Perm Reagent (Invitrogen, USA), followed by intracellular staining the cells with PE-labeled anti-IL-17A. For analysis of Treg cells, PBMC were collected into tubes without PMA and ionomycin stimulation, surface stained for anti-CD4 and anti-CD25 at 4 °C for 30 min and treated with the Fix and Perm Reagent, followed by intracellular staining with PE/Cy5.5-labeled anti-Foxp3 following the manufacturer’s protocol (eBioscience, USA). After washing, the cells were resuspensed with staining buffer (eBioscience, USA), and analyzed by flow cytometry with a FACS Calibur (BD FACSCanto™ II, USA), and the results were analyzed with Cell Quest software (BD Biosciences, USA).

### Enzyme-linked immuno sorbent assay (ELISA)

Serum from AAU patients and healthy controls were collected and stored at −80 °C. ELISA for IL-17, TGF-β, IL-6 and IL-23 were performed using kits from R&D according to the manufacturer’s instructions.

### Quantitative real-time RT-PCR analysis

Total RNA was extracted from PBMC by using Trizol reagent (Invitrogen, Carlsbad, CA, USA). cDNA was prepared by reverse transcription with oligo (dT) from total RNA extraction (TaKaRa, Japan). Real-time PCR for Foxp3, ROR-γt and a reference gene (β-actin) was performed using an ABI 7500 Sequence Detection System (Applied Biosystems, USA) with the SYBR green master mix kit (Qiagen, Hilden, Germany). β-actin was used as an endogenous reference with primers: 5′-TGGACTTCGAGCAAGAGATG-3′ (forward primer) and 5′-GAAGGAAGGCTGGAAGAGTG-3′ (reverse primer). The primer sequences for Foxp3 were 5′-GTGGCCCGGATGTGAGAAG-3′ (forward primer) and 5′-GGAGCCCTTGTCGGATGATG-3′ (reverse primer). The primer sequences for ROR-γt were 5′-CCTGGGCTCCTCGCCTGACC-3′ (forward primer) and 5′-TCTCTCTGCCCTCAGCCTTGCC-3′ (reverse primer). The primer sequences for IL-17A were 5′-CGGCTGGAGAAGATACTGGT-3′ (forward primer) and 5′-TTAGTCCGAAATGAGGCTGTC-3′ (reverse primer). The primer sequences for IL-17F were 5′-CACCAAGGCTGCTCTGTTTC-3′ (forward primer) and 5′-GCACCTCTTACTGCACATGGT-3′ (reverse primer). The primer sequences for IL-23 were 5′-GCAGAGATTCCACCAGGACT-3′ (forward primer) and 5′-AGCAGCAACAGCAGCATTAC-3′ (reverse primer). The primer sequences for IL-6 were 5′-ACAGACAGCCACTCACCTCTT-3′ (forward primer) and 5′-TTTCAGCCATCTTTGGAAGG-3′ (reverse primer). The primer sequences for TGF-β were 5′-ACCTTGGGCACTGTTGAAGT-3′ (forward primer) and 5′- CTCTGGGCTTGTTTCCTCAC-3′ (reverse primer). The Ct values of Foxp3, ROR-γt, IL-17A, IL-17F, IL-23, IL-6 and TGF-β were normalized against those of β-actin. Data are presented relative to the normalized Foxp3 and ROR-γt levels.

### Statistical analysis

All data were expressed as mean ± s.e.m. Student’s t-test were used for the determination of statistical significance, and a *P* value of <0.05 or less indicated the presence of a statistically significant difference. Spearman’s rank correlation was used to analyze the relationships between the expression of Thl7, Treg and the ratio of Th17/Treg in PBMC of AAU patients and disease activity score. All analysis was performed using version SPSS16.0 software (SPSS Inc., USA).

## Additional Information

**How to cite this article**: Zhuang, Z. *et al*. Imbalance of Th17/Treg cells in pathogenesis of patients with human leukocyte antigen B27 associated acute anterior uveitis. *Sci. Rep.* **7**, 40414; doi: 10.1038/srep40414 (2017).

**Publisher's note:** Springer Nature remains neutral with regard to jurisdictional claims in published maps and institutional affiliations.

## Supplementary Material

Supplementary Information

## Figures and Tables

**Table 1:** General Characteristics of the Study Participants.

	Health controls n = 20	HLA-B27 AAU patients n = 20
Age (yrs, mean ± sd)	24–50 (39.8 ± 6.9)	27–50 (38.5 ± 6.2)
male sex (%)	70	70
disease activity score	0	5.15 ± 2.13
