# Supplementary material for: Imbalance of Th17/Treg cells in pathogenesis of patients with human leukocyte antigen B27 associated acute anterior uveitis
Source: Sci Rep. 2017 Jan 16;7:40414. doi: 10.1038/srep40414 (PMC5238419; doi:10.1038/srep40414)
Supplement: Supplementary Information [file srep40414-s1.doc]

**Imbalance of Th17/Treg cells in pathogenesisof patients with human leukocyte antigen B27 associated acute anterior uveitis**

Zhenchao Zhuang^1,2+^, Yuqin Wang^3+^, Gejing Zhu^1,2^, Yunfeng Gu^1^, Liping Mao^1^, Meng Hong^2^, Yali Li^1^, Meiqin Zheng^1,2*^

^1^Division of Clinical Lab, Eye Hospital of Wenzhou Medical University, Wenzhou 325035, Zhejiang, China

^2^School of Laboratory Medicine and Life Science, Wenzhou Medical University, Wenzhou 325025, Zhejiang, China

^3^Department of Ophthalmology, Eye Hospital of Wenzhou Medical University, Wenzhou 325035, Zhejiang, China

^+^These authors contributed equally to this work and should be considered co-first authors

Correspondence and requests for materials should be addressed to M.Q.Z. (email: [zmqlyllh@126.com](mailto:zmqlyllh@126.com); TEL: 86-577-88068857)

**Supplementary Data**

**Comparison between the expression of CD4^+^** **IFN-γ^+^, CD4^+^IL-4^+^ and the IFN-γ and IL-17A double expressing Th17 cells in peripheral blood**

According to flow cytometric analysis, the percentage of CD4^+^IFN-γ^+^ and CD4^+^IL-4^+^T cells in peripheral blood of patients with HLA-B27-positive AAU (14.35%±1.48, ranged 9.17%-21.4%) (3.58%±0.35, ranged 2.26%-5.86%) increased, compared with that of the control group (11.07%±0.87, ranged 6.57%-14.9%) (3.40%±0.33, ranged 1.78%-5.40%) (P=0.085, P=0.719) (Supplementary FigS1.Top left and right, Supplementary FigS1.Centre left and right), but there were no statistically significant difference between the two groups. The IFN-γ and IL-17A double expressing Th17 cells in peripheral blood of patients with HLA-B27-positive AAU (0.22%±0.03, ranged 0.08%-0.39%) significantly increased, compared with that of the control group (0.11%±0.02, ranged 0%-0.21%) (P<0.05) ( Supplementary FigS1.Bottom left and right).


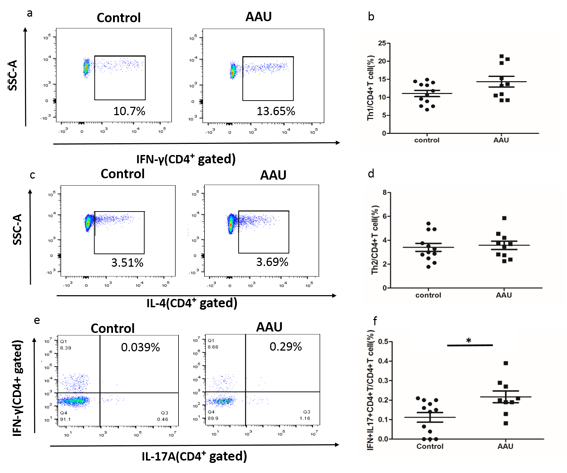
Supplementary Fig.S1 The distribution of CD4^+^IFN-γ^+^ cells, CD4^+^IL-4^+^cells and the IFN-γ and IL-17A double expressing Th17 cells in peripheral blood of patients with HLA-B27-associated AAU and controls. **a-b，**CD4^+^IFN-γ^+^ cells in control group and patients with HLA-B27-associated AAU; **c-d,** CD4^+^IL-4^+^cells in control group and patients with HLA-B27-associated AAU; **e-f,** The IFN-γ and IL-17A double expressing Th17 cells in control group and patients with HLA-B27-associated AAU. Data represent means±SDs.Data were analyzed using Student’s t-test. Error bars represent s.e.m. *, P < 0.05; **, P <0.01; ***, P < 0.001 each control group vs HLA-B27-associated AAU patients. (AAU= acute anterior uveitis)
